# Fate of the Fc fusion protein aflibercept in retinal endothelial cells: competition of recycling and degradation

**DOI:** 10.1007/s00417-018-4166-7

**Published:** 2018-10-26

**Authors:** Heidrun L. Deissler, Gerhard K. Lang, Gabriele E. Lang

**Affiliations:** Department of Ophthalmology, University Hospital of Ulm, Prittwitzstrasse 43, 89075 Ulm, Germany

**Keywords:** Retinal endothelial cells, Aflibercept, Fc fusion protein, IgG, Neonatal Fc receptor, Recycling, Degradation

## Abstract

**Purpose:**

Intravitreal injection of the VEGF-binding protein aflibercept is widely used to treat various ocular diseases. In vitro, immortalized bovine retinal endothelial cells (iBREC) take up and transport aflibercept through the cell layer in a serum-dependent manner, likely mediated through the neonatal Fc receptor (FcRn), but degradation of the Fc domain-containing protein might be a competing intracellular process. Therefore, aflibercept’s associations with proteins either involved in FcRn-mediated transport or in the lysosomal pathway were studied.

**Methods:**

Confluent iBREC pre-cultivated with or without FBS were exposed for 4 h to in vivo achievable 250 μg/ml aflibercept, before cells were harvested for immunofluorescence staining or preparation of protein extracts. Intracellular localization of aflibercept and putative co-localizations with proteins involved in transport of IgG/FcRn complexes, i.e., endosomal Rab4 and Rab11, components of the cytoskeleton, motor proteins, or with marker proteins characteristic of multivesicular bodies or lysosomes were assessed by co-immunofluorescence stainings. Amounts of expressed endogenous proteins and of internalized aflibercept were determined by Western blot analyses.

**Results:**

Aflibercept-specific perinuclear staining overlapped with that of the motor protein dynein whereas double staining with an anti-kinesin antibody resulted in a patchy pattern. In addition, aflibercept was typically present close to microtubules and often co-localized with α-tubulin. Rab4 and Rab11 stainings partly overlapped with the perinuclear staining of aflibercept whereas co-localization with Rab7 (in late endosomes/lysosomes) was only rarely seen. Interestingly, aflibercept but not the IgG bevacizumab broadly co-localized with the cation-independent mannose 6-phosphate receptor characteristic of multivesicular endosomes. In accordance with partial degradation beside transcytosis, the amount of intracellular aflibercept increased when cells were treated with protease inhibitors MG-132 or MG-101. Serum-deprived iBREC expressed less Rab11 and dynein but slightly more Rab4.

**Conclusion:**

After uptake by iBREC, aflibercept is present in organelles associated with FcRn-mediated transport, but part of the protein is subject to degradation. Transport inhibition of aflibercept during cultivation without FBS is likely a consequence of an attenuated exocytosis due to decreased expression of Rab11.

## Introduction

Aflibercept, a recombinant protein binding to members of the family of vascular endothelial growth factors (VEGFs), is widely used to treat various ocular diseases associated with an elevated VEGF expression [1, 2]. After intravitreal injection into the mammalian eye, aflibercept, which contains an Fc (fragment crystallizable) domain, was detected in ocular structures including retinal vessels and the retinal pigment epithelium (RPE), implying at least partial clearance of aflibercept via the posterior route [3–5]. Accordingly, retinal endothelial cells (REC) or cells of the RPE take up aflibercept in vitro, and transcellular transport through these types of cells was observed [6–8]. A carrier protein likely involved in shuttling IgG and other Fc-containing polypeptides is the neonatal Fc receptor/transporter (FcRn), which indeed is expressed in various parts of the eye including the retinal endothelium and cultivated REC [9–12]. In human dermal microvascular endothelial cells (HMEC), the FcRn is present in early endosomes characterized by presence of early endosome antigen 1 (EEA1) and the small Ras-like GTPase 4 (Rab4) [13–15]. In these organelles, it binds at low pH to IgG that has been internalized by pinocytosis, after which the complex is separated from unbound IgG and other proteins in sorting endosomes (positive for Rab4 and Rab11) [9, 13–15]. FcRn-bound IgG is then transported by recycling exosomes (positive for Rab11 but not for Rab4) to be released at the cell surface, thereby avoiding subsequent intracellular degradation in lysosomes [13, 15, 16]. In contrast, unbound IgG or other proteins destined to be eventually degraded in lysosomes, could be detected at an earlier stage mainly in multivesicular endosomes characterized by the presence of the cation-independent mannose 6-phosphate receptor (CI-M6PR) [17].

Because aflibercept is a protein with an Fc terminus of a human IgG1, a role of the FcRn in its transport through REC is a plausible assumption, recently supported by our investigations with immortalized bovine REC (iBREC). Through a monolayer of these cells, aflibercept is transported via an FcRn-mediated process, which was also observed in similar experiments with the VEGF-A-binding humanized IgG bevacizumab [7, 12]. However, aflibercept as an Fc fusion protein has distinct properties different from those of a classical IgG and this might result in at least partly altered mechanisms of intra-REC transport. Fc fusion proteins indeed tend to have a shorter serum half-life compared with IgG often due to a lower binding affinity to the FcRn [18, 19]. Therefore, we investigated whether aflibercept taken up by iBREC was co-localized with proteins involved in an FcRn-mediated transport or those indicative of the lysosomal route. The human and bovine homologs of proteins likely participating in the investigated processes, i.e., FcRn, Ras-like GTPases, CI-M6PR, and others, are highly conserved or even identical, allowing experiments based on the well-established model of iBREC and double immunofluorescence staining with available specific antibodies [20, 21]. It was also directly confirmed in several studies that *human* IgG is efficiently bound, transported, and released by *bovine* FcRn in vivo and in vitro [22–24].

## Material and methods

### Aflibercept and antibodies

Bayer Vital GmbH (Leverkusen, Germany) kindly provided Eylea (40 mg/ml aflibercept in 10 mM sodium phosphate, 40 mM NaCl, 0.03% polysorbate 20, 5% sucrose, pH 6.2) [1, 2]. The humanized anti-VEGF antibody bevacizumab (Avastin; 25 mg/ml in 50 mM sodium phosphate, 6% α,α-trehalose dihydrate, 0.04% polysorbate 20, pH 6.2; Roche Pharma, Grenzach-Wyhlen, Germany) was repackaged at the pharmacy of the University Hospital Ulm and provided in syringes, which were stored at 4 °C not longer than 4 weeks [25]. Tables 1 and 2 provide all relevant information on primary and secondary antibodies used for immunofluorescence staining or Western blot analyses.

**Table 1:** Primary antibodies used

Target	Host	Type	Source	Working concentrations
Actin	Mouse	Monoclonal	Clone AC-40, Abcam (Cambridge, UK), ab11003	WB, 500 ng/ml
Caveolin-1	Rabbit	Polyclonal	Abcam, ab2910	WB, 20 ng/ml; IF, 1 μg/ml
CI-M6PR/IGF2-R	Mouse	Monoclonal	Thermo Fisher Scientific (Langenselbold, Germany), MA1-066	IF, 2 μg/ml
Claudin-1	Rabbit	Polyclonal	JAY.8, Thermo Fisher Scientific, 51-9000	WB, 1 μg/ml
Claudin-5	Rabbit	Polyclonal	Thermo Fisher Scientific, 34-1600	WB, 100 ng/ml
Dynein (intermediate chain 1, IC74)	Mouse	Monoclonal	Clone 74.1, Abcam, ab23905	WB, 1 μg/ml; IF, 10 μg/ml
EEA1	Rabbit	Polyclonal	Abcam, ab137403	WB, 70 ng/ml; IF, 20 μg/ml
FcRn (large chain)	Rabbit	Polyclonal	Bio-techne (Wiesbaden, Germany), NBP1-89127	WB, 3 μg/ml
Kinesin (heavy chain)	Mouse	Monoclonal	Clone SUK-4, Abcam, ab28060	IF, 1.3 μg/ml
Rab4	Rabbit	Polyclonal	Thermo Fisher Scientific, PA3-912	WB, 1:500; IF, 1:75
Rab7	Rabbit	Polyclonal	Thermo Fisher Scientific, PA5-22959	IF, 1:100
Rab11a, Rab11b, Rab11c	Goat	Polyclonal	Sicgen Antibodies (Carcavelos, Portugal), AB3035	WB, 1.5 μg/ml
Rab11	Rabbit	Polyclonal	Abcam, ab3612	IF, 5 μg/ml
α-Tubulin	Mouse	Monoclonal	Clone DM1A, Abcam, ab7291	WB, 200 ng/ml; IF, 1 μg/ml
Vimentin	Mouse	Monoclonal	Clone RV202, Abcam, ab8978	WB, 70 ng/ml; IF, 1 μg/ml

*WB*, Western blot analyses; *IF*, immunofluorescence stainings; *CI-M6PR/IGF-2R*, cation-independent mannose 6-phosphate receptor/insulin-like growth factor 2 receptor; *EEA1*, early endosomal antigen 1

**Table 2:** Secondary antibodies used

Target	Host	Type	Conjugate	Source	Working concentrations
IgG, γ-chain, human	Goat	Polyclonal	Coupled to HRP	Thermo Fisher Scientific, 628,420	WB, 1:5000
Whole IgG, rabbit	Goat	Polyclonal	Coupled to HRP	Biorad (Munich, Germany), 170-5046	WB, 1:30,000
Whole IgG, mouse	Goat	Polyclonal	Coupled to HRP	Biorad, 170-5047	WB, 1:30,000
IgG, H+L chains, goat	Donkey	Polyclonal	Coupled to HRP	Bio-techne, HAF109	WB, 1:4000
IgG, H+L chains, human	Goat	Polyclonal	Coupled to AlexaFluor594	Thermo Fisher Scientific, A11014	IF, 1:500
IgG, H+L chains, rabbit	Goat	F(ab’)_2_ fragment	Coupled to AlexaFluor488	Thermo Fisher Scientific, A11070	IF, 1:500
IgG, H+L chains, mouse	Goat	F(ab’)_2_ fragment	Coupled to AlexaFluor488	Thermo Fisher Scientific, A11017	IF, 1:500

*HRP*, horseradish peroxidase; *WB*, Western blot analyses; *IF*, immunofluorescence stainings

### Treatment of iBREC with effectors

Generation and characterization of telomerase-immortalized microvascular endothelial cells from bovine retina (iBREC), which even after extended cultivation show the cobblestone-like morphology typical of EC-forming retinal capillaries, were comprehensively described [12, 21, 26]. Cells were cultivated on fibronectin-coated (Corning, Amsterdam, The Netherlands) surfaces in Endothelial Cell Growth Medium MV (ECGM; Promocell, Heidelberg, Germany) containing 1 g/l glucose, 0.4% Endothelial Cell Growth Supplement/H, 90 μg/ml heparin, 10 ng/ml human epidermal growth factor (hEGF), 100 nM 11*β*-hydroxycortisone, and 5% fetal bovine serum (FBS) as described previously [12, 21]. Cells were used from passages 25 to 60 counting from the stage of primary culture for which we had confirmed stable expression of marker proteins typical for EC (e.g., von Willebrand factor, tight junction-proteins claudin-1 and claudin-5) and proteins under investigation [6, 7, 12, 21]. Pericytes or other cells expressing α-smooth muscle actin are not present in iBREC cultures [21]. To ensure authenticity and optimal conditions of iBREC in the experiments, we routinely recorded their characteristic proliferation profile by electric cell-substrate impedance measurements using the microelectronic biosensor system for cell-based assays xCELLigence RTCA DP (Acea, OLS, Bremen, Germany) [27]. All experiments—repeated at least twice—were performed with confluent monolayers of iBREC formed after cultivation for 4 days. In control experiments, we processed the cells identically in medium only lacking the effector(s) investigated.

Prior to investigations with confluent iBREC, ECGM was replaced with SHM (same medium as ECGM but lacking hEGF) or—to study the effect of FBS—with SFM (same as SHM but without FBS) for 1 day. Aflibercept (or bevacizumab) was added for additional 4 h before cells were harvested for preparation and analyses of subcellular fractions or fixed for immunofluorescence staining (see below). VEGF-binding proteins were used at a final concentration of 250 μg/ml, resembling the in vivo situation after intravitreal injection [2, 6, 7, 12].

To assess the effects of the protease inhibitors MG-101 or MG-132 (Selleckchem, Absource Diagnostics, Munich, Germany) on barrier stability, confluent iBREC grown on gold electrodes (E-Plates 16 PET, Acea) were treated with these substances at concentrations ranging from 20 nM to 5 μM or to 20 μM, respectively [27–30]. The cell index as a measure of permeability was recorded every 5 min for at least 24 h [27].

### Preparation of protein extracts

Fresh or frozen cell pellets were subjected to subsequently carried out extraction steps with different buffers (ProteoExtract Subcellular Proteome Extraction Kit, Merck Millipore, Darmstadt, Germany), resulting in subcellular fractions containing proteins localized in the cytoplasm, in membranes/organelles, or components of the cytoskeleton [7, 12]. Protein concentrations in the samples were determined using the Pierce Thermo Scientific BCA Protein Assay Kit according to the manufacturers’ instructions.

### Western blot analyses and imaging

For Western blot analyses, proteins (3 μg/lane) were separated by SDS-polyacrylamide electrophoresis under reducing conditions followed by electroblotting as described in detail previously [7, 27]. Appropriate IgG-horseradish peroxidase conjugates (Table 2) and the chemiluminescence kit Pierce ECL Plus Western Blotting Substrate (Thermo Fisher Scientific) were used to detect antigen-bound primary antibodies. As previously observed, the horseradish peroxidase conjugate used to detect human IgG gave rise to an additional unspecific signal when isolated cytoskeleton proteins were analyzed [6, 7, 12]. Chemiluminescence signals from the so processed membranes were directly scanned with the imaging system Fusion Pulse TS (Vilbert Lourmat, VWR, Darmstadt, Germany) resulting in images with bright specific bands contrasting with a dark background. To quantify the signals, peak volumes of the corresponding bands (> 4 replicates) determined with EvolutionCapt software (Vilbert Lourmat) were standardized in relation to those obtained from similarly processed control cells.

### Double immunofluorescence staining

Confluent iBREC were cultivated on fibronectin-coated two-chamber slides (x-well PCA Tissue Culture Chambers; Sarstedt, Nuembrecht, Germany) and exposed to aflibercept or bevacizumab for 4 h as described above [7, 12]. After methanol-fixation (10 min at − 20 °C), cells were permeabilized for 10 min in 0.25% Nonidet P-40 substitute (Roche, Mannheim, Germany) diluted in PBS without Ca^2+^/Mg^2+^ ions (PBSd). After incubation of the slides in blocking solution (10% ImmunoBlock, Roth, Karlsruhe, Germany) for 60 min at room temperature, they were treated for 30 min with AlexaFluor594-conjugated antibodies binding to human IgG to detect aflibercept or bevacizumab. Slides were then exposed to primary antibodies recognizing endogenous proteins of interest for 1 h at room temperature, and subsequently to appropriate AlexaFluor488-conjugated goat F(ab’)_2_ fragments for 30 min. Primary and secondary antibodies were always diluted in 1% ImmunoBlock/PBSd. For examination by fluorescence microscopy (DM4000B, FW4000, Leica, Wetzlar, Germany), cells were embedded in ProLong Gold/Diamond Antifade Mountant with DAPI (Thermo Fisher Scientific) [7, 12, 27].

### Transcytosis assays

Transcytosis assays were performed as published to assess transport of aflibercept through a confluent iBREC monolayer cultivated on membrane inserts (4.7 cm^2^, pore size 0.4 μm; Corning) from the lower to the upper chamber [7, 12]. After incubating iBREC for 1 day in SHM or SFM, aflibercept (final concentration, 250 μg/ml) was placed in the bottom chamber and samples were then taken from the upper chamber at indicated time points. Presence of aflibercept in these samples was assessed by Western blot analyses and peak volumes of the corresponding bands (four replicates) determined with EvolutionCapt software (Vilbert Lourmat) were normalized in relation to those obtained from 1 ng aflibercept.

### Statistical analyses

Mann-Whitney tests or one-way analyses of variance (ANOVA) followed by Tukey’s test were used to compare differing groups of quantified antigen-specific signals from Western blot analyses (Graph Pad Prism 6; Graph Pad Software, San Diego, USA), and differences resulting in *p* values below 0.05 were considered significant. Means and standard deviations were provided as numbers or in scatter plots.

## Results

### Aflibercept was present in organelles associated with FcRn-mediated transport and in multivesicular endosomes within 4 h after uptake

To determine aflibercept’s intracellular localization, we performed double-immunofluorescence stainings of iBREC treated with pharmacologically achievable 250 μg/ml of the therapeutic protein for 4 h. Then aflibercept had been taken up and transported through an iBREC monolayer, and substantial amounts of the recombinant protein remained in the cells resulting in a pronounced perinuclear staining in addition to a faint and diffuse overall intracellular staining [6, 7]. Direct targeting the FcRn was not possible because commercially available antibodies failed to give specific signals, but the assessed proteins were reported to be crucially involved either in the transport of FcRn/IgG-complexes or in lysosomal processes in endothelial cells (EC) or other cell types [7, 11–17, 31–34].

**Fig. 1:**
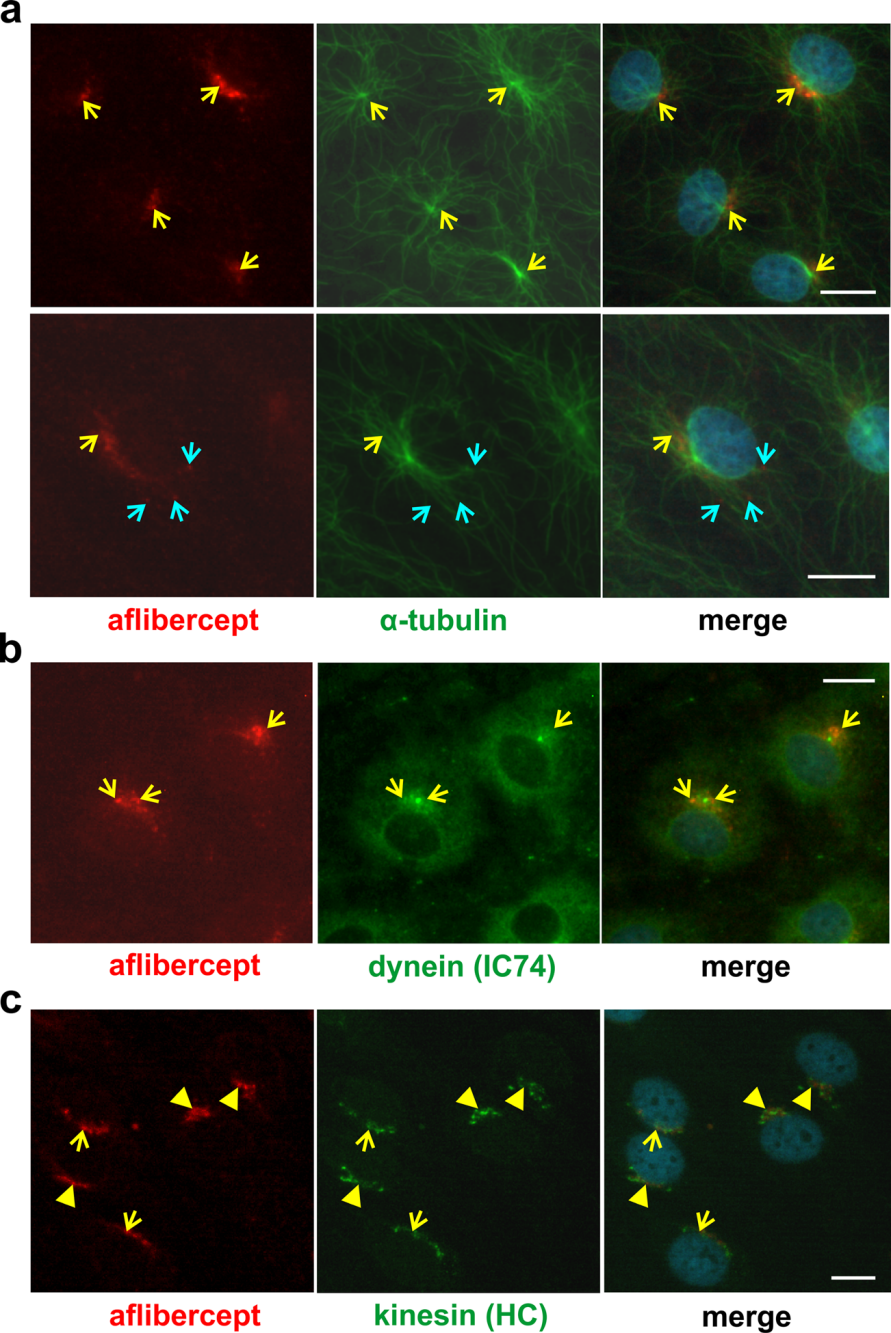
Aflibercept is co-localized with α-tubulin and dynein. Confluent iBREC treated with aflibercept were immunostained to detect aflibercept (red), α-tubulin (**a**, green), dynein intermediate chain IC74 (**b**, green), or kinesin heavy chain (HC, **c**, green). **a** α-Tubulin fibers originating from the microtubule organization center stretched to the periphery of the cells. Aflibercept-specific signals overlapped with those of α-tubulin at the microtubule organization center (yellow arrows). They were also close to the α-tubulin fibers in the cell periphery (blue arrows). **b** Dynein signals around the nucleus overlapped with those of aflibercept (yellow arrows). **c** The strong perinuclear kinesin-specific staining and that of aflibercept gave rise to a mosaic pattern (yellow arrowheads). Scale bar, 10 μm

**Fig. 2:**
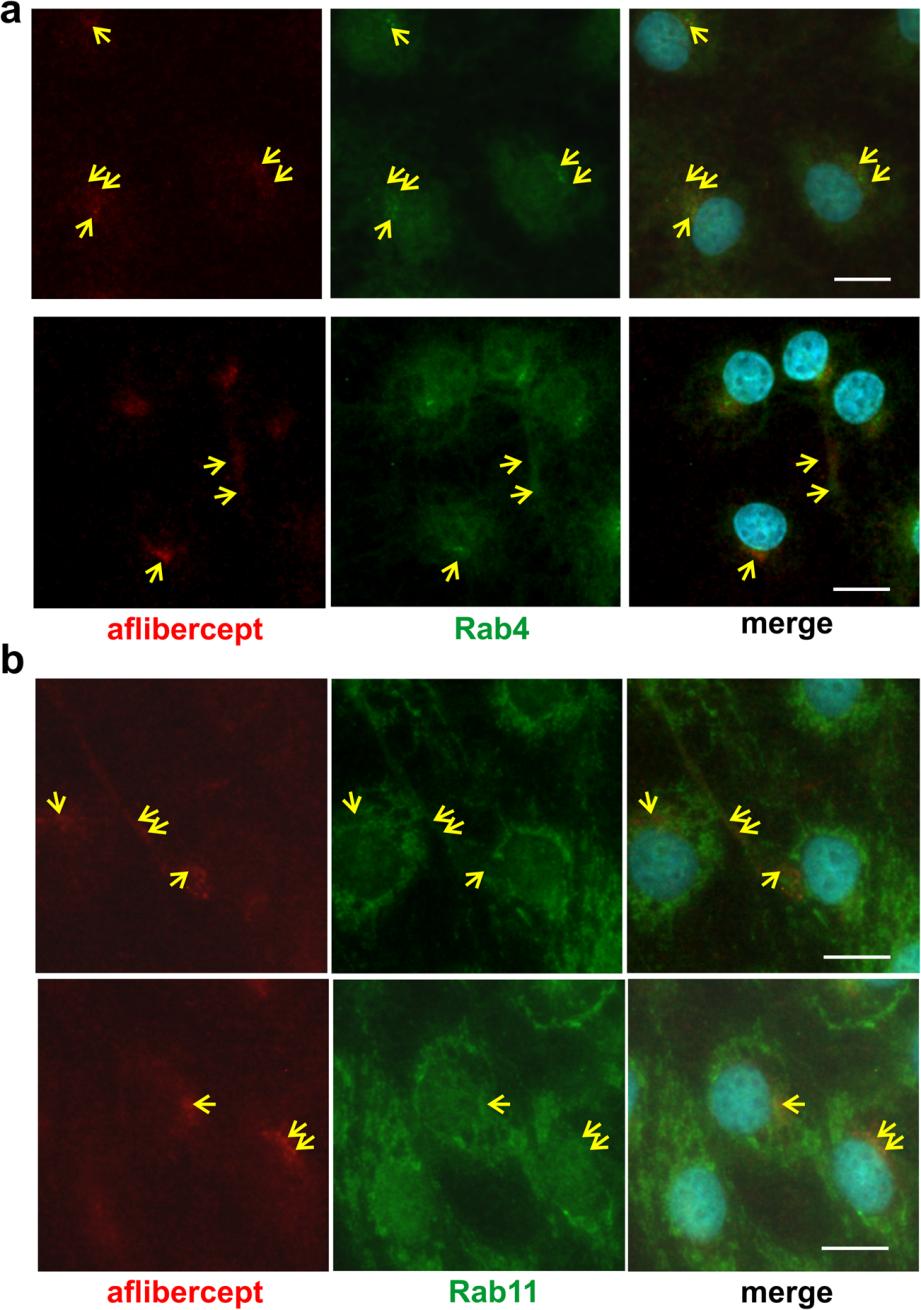
Aflibercept is co-localized with Rab4 and Rab11. Aflibercept (red), Rab4 (**a**, green), or Rab11 (**b**, green) were visualized by double immunofluorescence staining after exposure of iBREC to the Fc fusion protein. Antibodies specific for Rab4 (**a**) or Rab11 (**b**) bound to a prominent perinuclear region stretching into the periphery of the cell with similar but not identical patterns. Overlapping of signals (yellow arrows) from Rab4 (**a**) or Rab11 (**b**) staining with those specific for aflibercept is most evident in the perinuclear region and partially seen in the peripheral areas. Scale bar, 10 μm

Microtubule are involved in the transport of IgG/FcRn complexes in various cell types [31–33]. Accordingly, aflibercept co-localized with the microtubule component α-tubulin mainly in the perinuclear region close to or identical with the microtubule organization center (Fig. 1a, yellow arrows) but also close to the tubulin fibers in the periphery of the cells (Fig. 1a, blue arrows). Members of the motor protein family of dyneins transport cargo along microtubules from the plasma membrane to the cell body whereas kinesins are involved in similar transport in the opposite direction [33]. Interestingly, aflibercept-specific staining overlapped with that of the intermediate chain (IC74) of dynein in the perinuclear region (Fig. 1b) whereas signals specific for the heavy chain (HC) of kinesin were rather patchy with only a few overlaps (Fig. 1c, yellow arrows or arrowheads). Co-localization of aflibercept with other cytoskeletal proteins, e.g., actin or vimentin, or with caveolin-1—a component of caveolae—was not observed. In HMEC, early endosomes (→ Rab4, EEA1), sorting endosomes (→ Rab4, Rab11), and recycling exosomes (→ Rab11) contain the FcRn or FcRn/IgG complexes [14, 15]. In accordance with our previous observation that the perinuclear staining of aflibercept often overlapped with that of EEA1, aflibercept was also co-localized in the perinuclear region with signals specific for Rab4 (Fig. 2a) [7]. In addition, stainings of aflibercept and Rab4 overlapped in the periphery of the cells. In the same subcellular regions, we also observed partial co-localization of aflibercept with Rab11 (Fig. 2b).

**Fig. 3:**
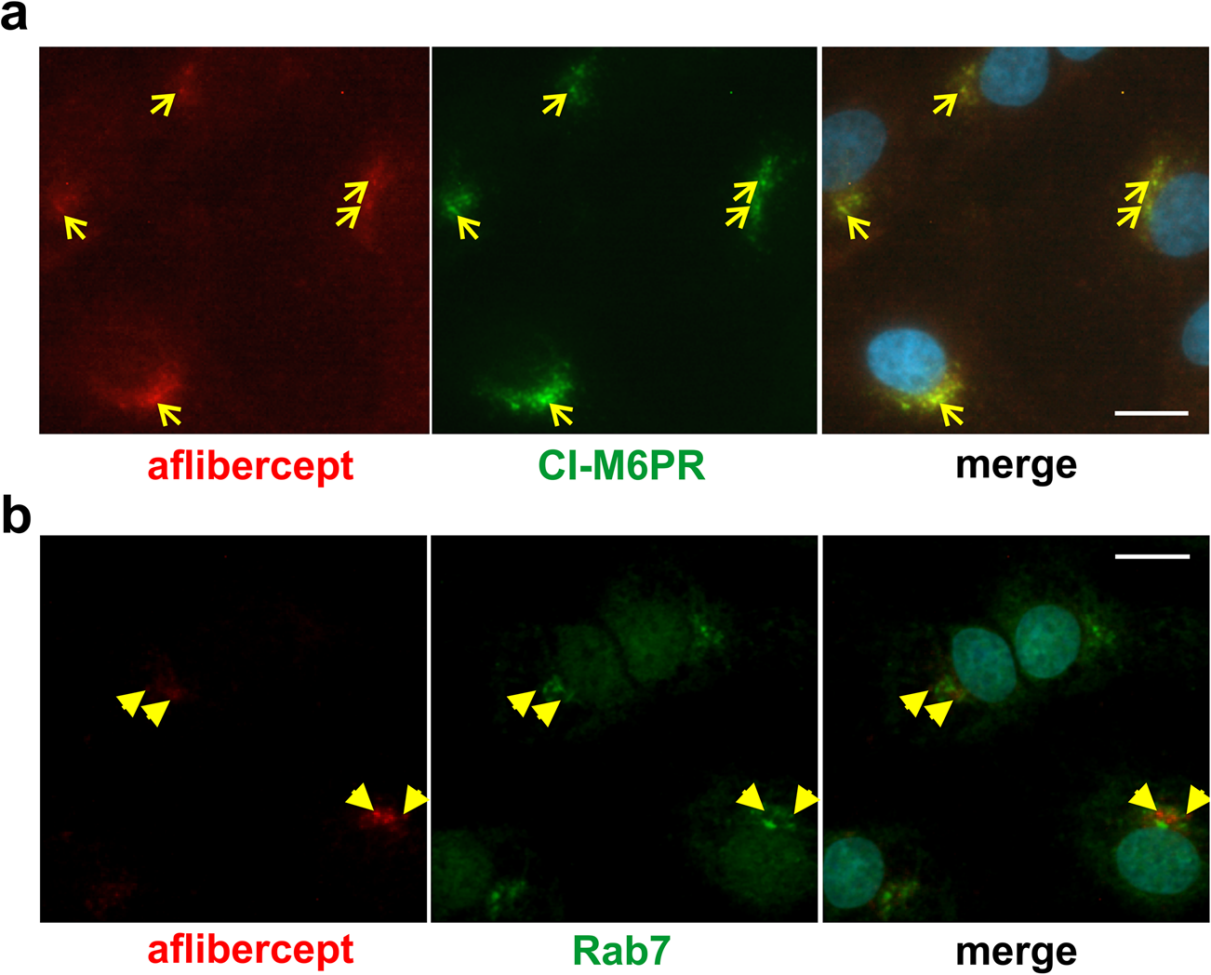
Aflibercept is largely co-localized with CI-M6PR. iBREC exposed to aflibercept were immunostained to detect aflibercept (red) and either the marker of multivesicular endosomes CI-M6PR (**a**, green) or Rab7 (**b**, green), specific for late endosomes and lysosomes. **a** The prominent CI-M6PR-specific staining close to the nucleus broadly overlapped with that of aflibercept (yellow arrows). **b** In contrast, aflibercept-specific signals and those specific for Rab7 gave rise to a mosaic pattern with few overlaps (yellow arrowheads). Scale bar, 10 μm

**Fig. 4:**
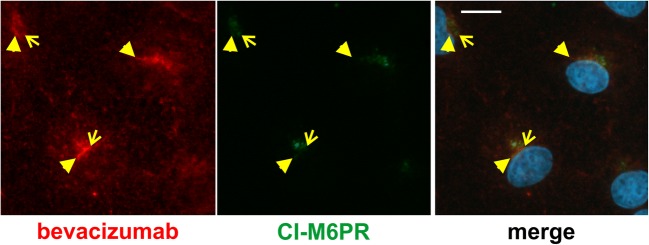
Bevacizumab is somewhat co-localized with CI-M6PR. After treatment of iBREC with the humanized IgG bevacizumab for 4 h, cells were immunostained to visualize the IgG (red) or CI-M6PR (green). The prominent CI-M6PR-specific signals close to the nucleus only to a small extent overlapped with those of bevacizumab (yellow arrowheads). Scale bar, 10 μm

Internalized aflibercept might be degraded, and proteins destined for degradation were detected in multivesicular endosomes, characteristically containing CI-M6PR involved in the transport of hydrolases from the trans-Golgi network to lysosomes [17]. Remarkably, the immunofluorescence stainings of aflibercept and CI-M6PR observed in the cells’ perinuclear regions widely overlapped (Fig. 3a). Co-localization of aflibercept with Rab7, a marker of late endosomes and lysosomes in EC, was seen sporadically and then as patchy signals rather than mixed colors (Fig. 3b) [34]. To rule out that presence of aflibercept in multivesicular endosomes might be the unlikely result of species incompatibility due to the interaction between the *human* Fc portion and *bovine* FcRn, we performed similar experiments with the VEGF-binding antibody bevacizumab also containing a human Fc terminus. As shown in Fig. 4 (yellow arrowheads or arrows), the immunofluorescence stainings specific for CI-M6PR and bevacizumab barely overlapped.

### Inhibition of proteasomal and lysosomal proteases increased the amount of intracellular aflibercept

**Fig. 5:**
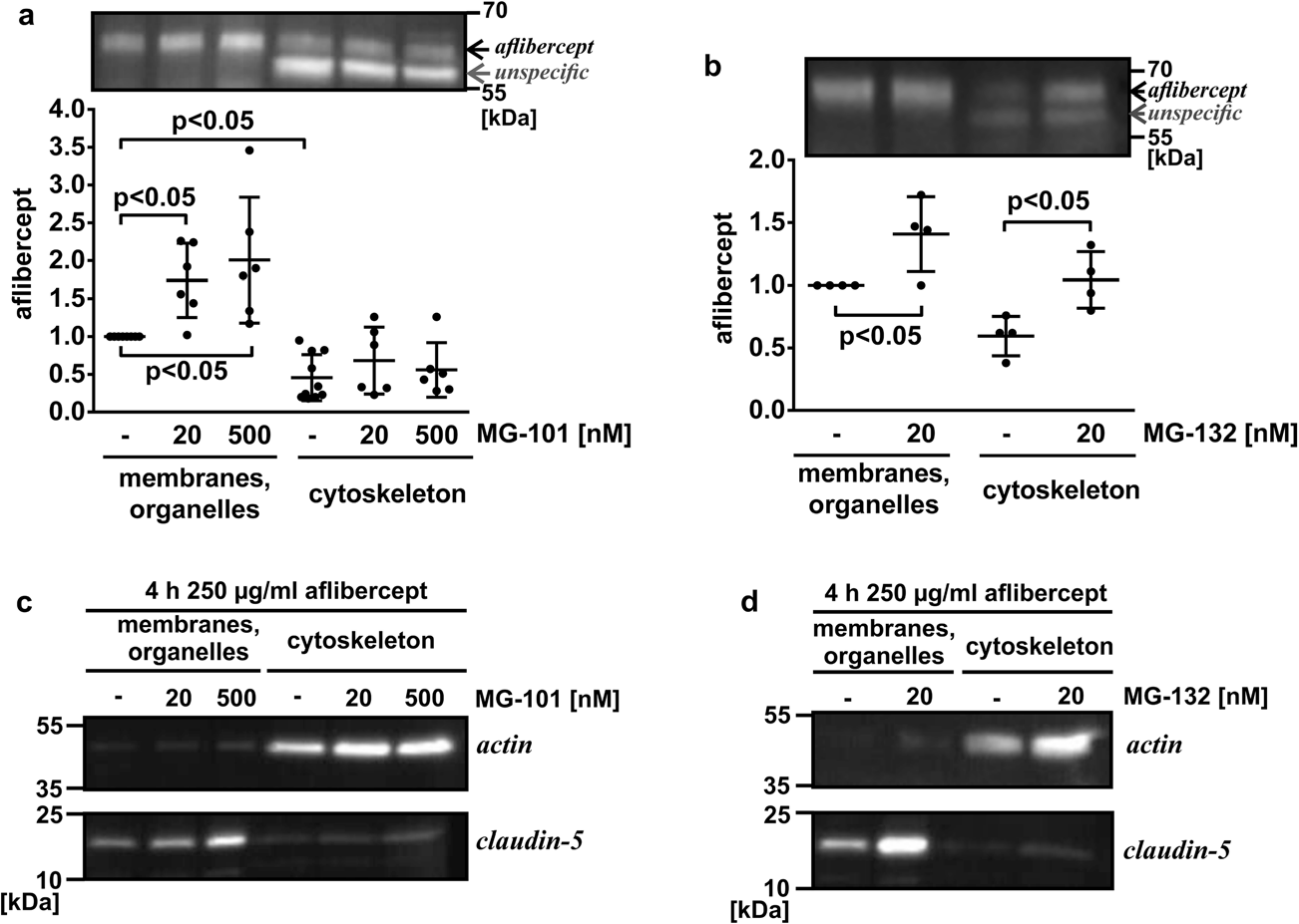
Inhibition of lysosomal or proteasomal proteases resulted in accumulation of aflibercept. After exposure of iBREC to aflibercept with or without inhibitors of lysosomal (**a**, **c** MG-101) or proteasomal (**b**, **d** MG-132) proteases, cells were harvested for preparation of subcellular fractions. In these, aflibercept (**a**, **b**), actin, or claudin-5 (**c**, **d**) were determined by Western blot analyses. To quantify the aflibercept-specific signals, peak volumes of the corresponding bands were measured and set in relation to those obtained from cells not exposed to the inhibitors. **a** The amount of aflibercept isolated with proteins from organelles and membranes was significantly higher after treatment with MG-101. **b** MG-132 also increased the amount of intracellular aflibercept. **c**, **d** Proteins from different subcellular compartments were efficiently separated without relevant cross-contamination: tight junction-protein claudin-5 (→ plasma membrane) was almost exclusively isolated together with proteins from membranes and organelles, and actin was only present in the fraction containing cytoskeleton proteins. Stronger signals in the lanes with samples from cells treated with the inhibitors MG-132 and MG-101 are due to the general inhibition of intracellular protein degradation

Our data support the hypothesis that the internalized aflibercept is transported through an iBREC monolayer in complex with FcRn, but part of the recombinant protein may be degraded. The cysteine protease inhibitor MG-101 counteracts the activity of lysosomal cathepsins L and B at a concentration of 20 nM or that of the non-lysosomal calpains I and II at 500 nM, respectively [28, 29]. Exposure of iBREC to either effective concentration of MG-101 together with aflibercept for 4 h resulted in a slight but significant increase of aflibercept isolated collectively with proteins from membranes and organelles (Fig. 5a). Because most of the intracellular protein destined to be degraded enters the ubiquitin-proteasome pathway, we pre-treated iBREC with 20 nM of the inhibitor MG-132 of proteasomal proteases, before aflibercept was added to the cells for 4 h [30]. Western blot analyses of proteins subsequently isolated from the membranes/organelles and the cytoskeleton revealed that more aflibercept accumulated during protease inhibition (Fig. 5b). As was to be expected, the amounts of endogenous proteins actin and claudin-5 were higher in samples isolated from cells treated with the inhibitors MG-101 or MG-132, confirming general inhibition of protein degradation pathways under these conditions (Fig. 5c, d). Higher concentrations of MG-101 or MG-132 could not be used because they resulted in barrier dysfunction and death of iBREC as recognized by cell index measurements.

### Serum depletion resulted in decreased expression of dynein and Rab11

**Fig. 6:**
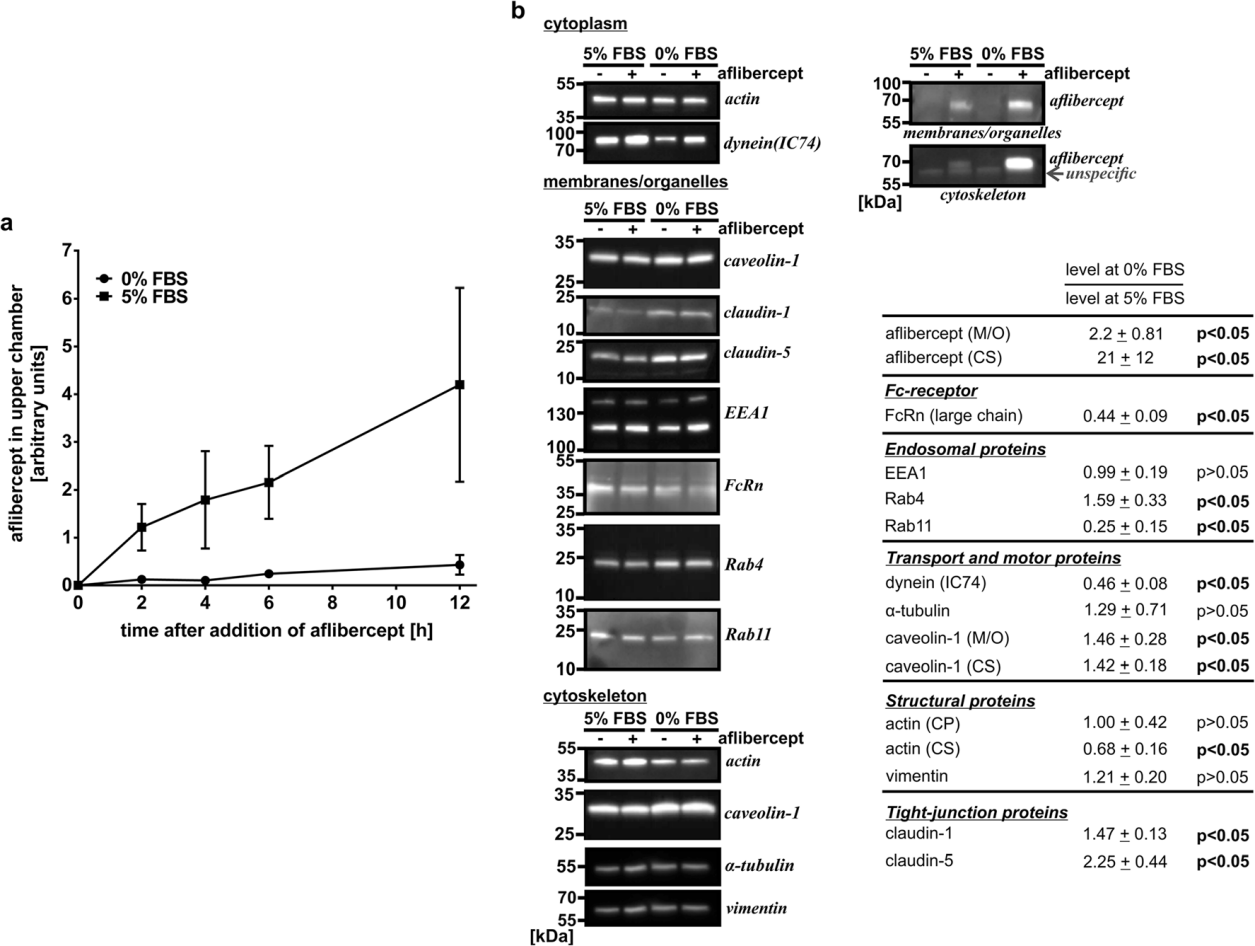
Cultivation of iBREC without FBS affected aflibercept’s transport rate and intracellular amount, as well as expression of proteins potentially involved its transport. **a** iBREC monolayers grown on porous membrane inserts were exposed to culture medium with or without FBS for 1 day. Aflibercept was then added to the lower chamber and its presence was assessed and quantified by Western blot analyses as described in materials and methods in samples taken from the upper chamber. Transport of aflibercept through iBREC was significantly faster in the presence of FBS. **b** After cultivation of iBREC with or without FBS for 1 day, aflibercept was added for additional 4 h. Then cells were harvested for preparation of subcellular fractions as indicated. Relative expression levels of proteins were determined by Western blot analyses as described in materials and methods. Cultivation without FBS resulted in significantly more internalized aflibercept and decreased expression of Rab11 and dynein, whereas amounts of other proteins were higher. M/O, membranes/organelles; CS, cytoskeleton; CP, cytoplasm

Aflibercept’s transport through an iBREC monolayer was considerably slower when cells were cultivated without FBS and this coincided with a significantly increased amount of the recombinant protein retained in the cells (Fig. 6a, b) [7]. We also determined the cell index as a measure of iBREC permeability and found that it was high and in the same range when iBREC were cultivated with or without FBS. This confirms the presence of a stable barrier preventing paracellular transport and allowing for transcellular transport only. Based on the assumption that aflibercept’s intracellular transport could be restricted as a consequence of a changed expression of involved proteins under these conditions, we analyzed their presence in iBREC treated for 4 h with the Fc fusion protein after having been cultivated with or without FBS for 1 day. Western blot analyses of proteins from the cytoplasm, membranes, or organelles and the cytoskeleton showed that presence of aflibercept did not significantly affect the levels of investigated proteins at either condition. Reduced expression of the FcRn after cultivation of iBREC under serum-free conditions was not due to a general decline in protein expression as some proteins investigated were not changed and others were even expressed significantly stronger, e.g., tight junction-proteins claudin-1 and claudin-5 (Fig. 6b). Interestingly, the levels of Rab11 and dynein were also lower whereas that of Rab4 was higher after cultivation of iBREC without FBS (Fig. 6b). In addition, cultivation conditions resulted in changes of the subcellular localization of Rab4 and to a lesser extent of Rab11. Both proteins were more focused in the perinuclear region in cells cultivated without serum and overlapping of the perinuclear stainings of Rab4 and aflibercept was then slightly more pronounced.

## Discussion

After its uptake by REC, aflibercept can be transported actively through the intracellular space but before its release from the cell surface at either side of the EC layer, it might enter the degradation pathway. That aflibercept has an Fc terminus suggests involvement of the FcRn in its transport and protection from proteolysis. To further elucidate aflibercept’s fate inside iBREC, we performed double immunofluorescence stainings with marker proteins either associated with an FcRn-mediated transport (→ EEA1, Rab4, Rab11) or with the lysosomal pathway in EC (→ CI-M6PR, Rab7) [13–17, 34]. We also included proteins of the cytoskeleton (→ α-tubulin, actin, vimentin) as well as motor proteins (→ dynein, kinesin) in our analyses because these might play a role in FcRn-mediated transport along microtubules [31–33]. Obtained data on potential co-localization help to reveal the fate of internalized aflibercept although these marker proteins do not specify a single pathway or subcellular compartment. Although immunofluorescence stainings result in fixed images showing the situation at one time point, they have the advantage over live cell imaging that normal conditions rather than overexpressed recombinant proteins modified with fluorescence tags are analyzed. We chose to investigate aflibercept’s localization in iBREC after its uptake in the absence of VEGF-A, because the tight barrier formed by the cells under these conditions only allowed transcellular transport without additional paracellular flow.

Aflibercept staining overlapped with or was close to that of α-tubulin, suggesting an active transport along the microtubules. This assumption is supported by the also observed co-localization of aflibercept and the dynein intermediate chain, which is part of the dynein motor protein complex carrying cargo from the plasma membrane to the cell body. Co-localization of aflibercept with Rab4 and Rab11 as well as with CI-M6PR, evident after 4 h of exposure, pointed to the vesicular containers in which the protein is transported. Presence of aflibercept in vesicles containing Rab4 and/or Rab11 is in accordance with previously observed localization in EEA1-presenting early endosomes and supports the hypothesis that the FcRn plays an important role in its transcellular transport [7]. Remarkably, aflibercept also seemed to be present in multivesicular endosomes of iBREC indicated by its proximity to the CI-M6PR. Whereas CI-M6PR^+^ multivesicular endosomes of HepG2 cells or of epithelial cells of the rat intestine contain the FcRn, it was not detected in such vesicles of HMEC [13, 32, 35]. However, a modified IgG not able to bind to the FcRn remained in sorting endosomes in HMEC, which then matured to multivesicular endosomes [13]. From this observation and our results, we conclude that unbound aflibercept molecules not interacting with the FcRn likely reach CI-M6PR^+^ multivesicular endosomes. It is a reasonable assumption that this is not due to an impaired interaction between the *human* Fc terminus of aflibercept and the *bovine* FcRn because the bovine receptor efficiently transports human IgG through cells [7, 12, 22–24]. Accordingly, the humanized VEGF-binding IgG bevacizumab was only rarely detected in multivesicular endosomes in iBREC (see Fig. 4).

An increased amount of aflibercept internalized by iBREC as a consequence of general inhibition of lysosomal and proteasomal proteases showed that at least part of the Fc fusion protein is indeed degraded. The observed partial overlap of aflibercept and Rab7 stainings supports this hypothesis as appearance of Rab7 indicates maturing of sorting endosomes to (Rab7^+^) late endosomes, which transfer proteins destined for degradation to lysosomes by tubule-mediated processes [36]. Aflibercept’s entering of a pathway leading to lysosomal degradation is in accordance with the reported lower serum half-lives of other Fc fusion proteins compared with monoclonal antibodies, likely caused by lower affinity to the FcRn [18]. A serum half-live of aflibercept significantly lower than that of bevacizumab was also confirmed by experiments in which the proteins were administered to monkey eyes by intravitreal injection [19]. The intracellular stability of the VEGF-A-binding Fab fragment ranibizumab taken up by iBREC is even lower than that of aflibercept [37]. This might be due to the lack of an Fc terminus necessary for binding by the FcRn which is protective against intracellular proteolysis. Accordingly, a lower serum half-life after intravitreal injection was also observed [19].

The assumption that FcRn, Rab11, Rab4, and dynein all play important roles in aflibercept’s transcellular transport is further supported by our observation that their expression or localization significantly changed when iBREC had been cultivated without FBS to slow down the transport process. Under all conditions investigated, iBREC established a strong barrier as confirmed by a high cell index, thereby allowing only transport through the cell layer. When iBREC were cultivated without FBS, the lower expression of FcRn, Rab11, and dynein was accompanied by an increased expression of other proteins including Rab4, and the accumulation of Rab4, Rab11, and EEA1 as well as aflibercept in the perinuclear region [7]. Interestingly, in HMEC sorting of an FcRn-IgG complex away from unbound IgG takes place in Rab4^+^/Rab11^+^ sorting endosomes which mature to Rab4^−^/Rab11^+^ recycling exosomes [15]. Considering these observations, decreased transport and accumulation of aflibercept in serum-deprived iBREC might be caused by shortage of Rab11^+^ recycling exosomes leading to attenuated exocytosis.

Studying the intracellular fate of IgG or related proteins like aflibercept in REC is of considerable importance because after their therapeutic intravitreal injection they quickly reach the pericytes and endothelial cells of retinal vessels. This was demonstrated for aflibercept, bevacizumab and Fc fragments, detected in retinal vessels 1 to 2 days after their intravitreal injection into the eyes of rodents and monkeys [3, 38–41]. The role of the Fc domain has been emphasized by showing that a VEGF trap derivative, which instead of the Fc terminus contains a dimerized coiled-coil domain, has an increased half-life after intravitreal injection into the rabbit eye compared with the normal VEGF trap with an Fc region [4]. Together with the observation that after intravitreal injection bevacizumab was present in REC of wild-type mice but not in those of FcRn^−/−^ mice, these findings strongly support the hypothesis that the Fc domain primarily destines the fate of an intravitreally injected protein and that the FcRn plays a crucial role in this process [39]. An FcRn-dependent transport of aflibercept through cells of the RPE was also observed [8]. In these cells, aflibercept is co-localized with the motor protein myosin 7a and with proteins of the cytoskeleton (→ actin), indicating a similar though not identical behavior of the Fc fusion protein in the two different cell types forming the outer and inner blood retina barrier.

**Fig. 7:**
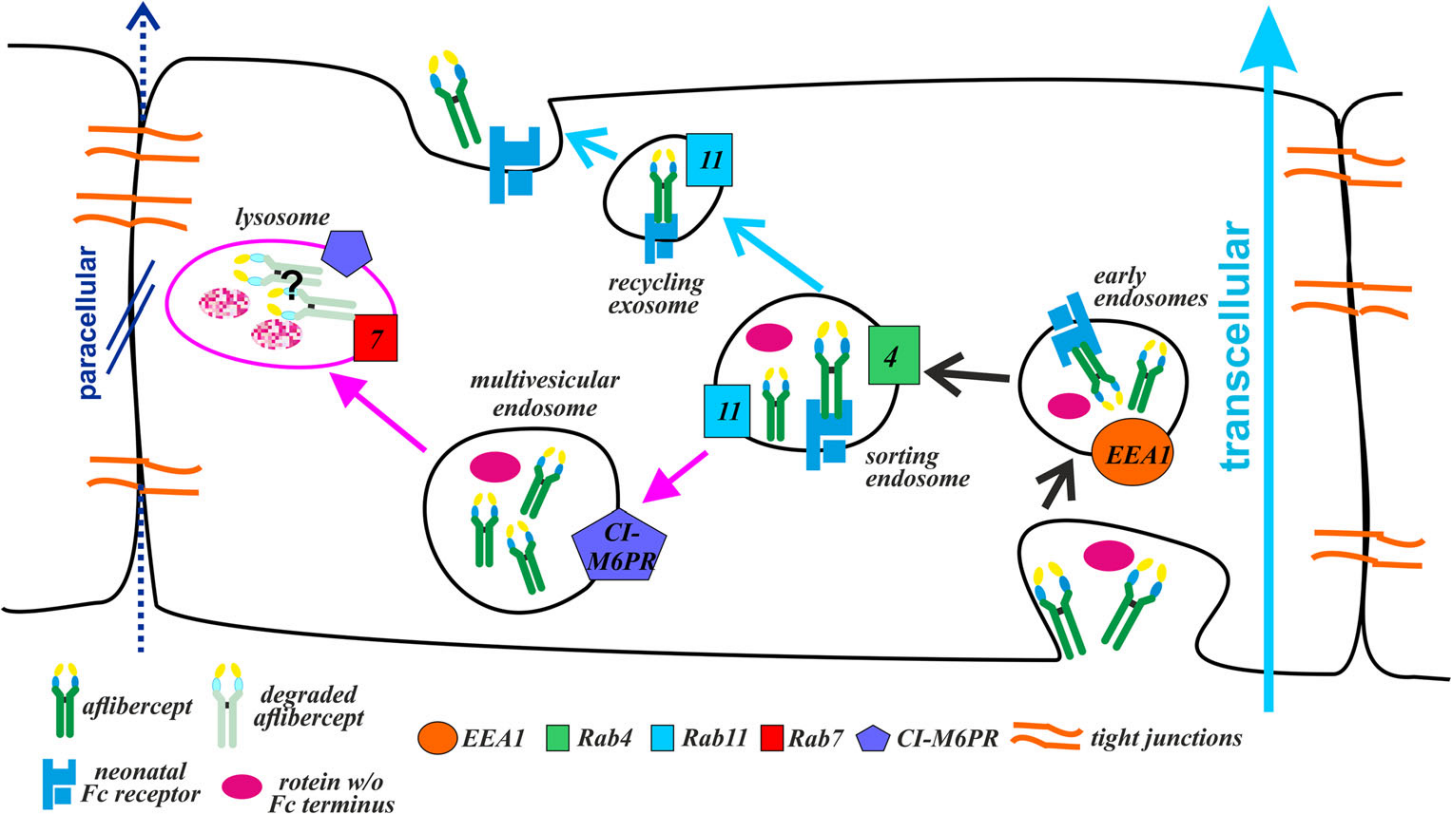
Cartoon proposing the fate of aflibercept after its uptake by iBREC. Within a few hours of exposure, aflibercept is distributed to EEA1^+^ early endosomes, endosomes positive for Rab4 and/or Rab11, and CI-M6PR^+^ multivesicular endosomes. This is in accordance with an FcRn-mediated transport of aflibercept through the intracellular space (light blue arrow), resulting in recycling of the Fc fusion protein at the surface of the cell. However, at least part of this protein is subject to degradation as indicated by our results on the effects of inhibitors of lysosomal or proteasomal proteases. Tight junctions closing the intercellular space prevent alternative paracellular transition (dark blue arrow) of the large aflibercept molecules

Summarizing, our results presented here are in accordance with the concept that aflibercept is transported through the intracellular space of REC in a process likely mediated by the FcRn as depicted in Fig. 7. Our previous findings that inhibition of the interaction between aflibercept and FcRn significantly slows down its transcellular transport also strongly support this hypothesis [7]. However, at least a fraction of the amount of internalized protein is subject to degradation, indicating that in detail the behavior of the Fc fusion protein inside REC is similar to though not identical with that of an IgG.
